# The Predictive Value of Emotion Regulation in Cocaine Use Disorder Severity: Psychotherapeutic Implications During Hospitalization for Detoxification

**DOI:** 10.1002/cpp.70155

**Published:** 2025-09-22

**Authors:** Alba Palazón‐Llecha, Joan Trujols, Mercè Madre, Santiago Duran‐Sindreu, Francesca Batlle, Núria Mallorquí‐Bagué

**Affiliations:** ^1^ Department of Psychology Universitat de Girona (UdG) Girona Spain; ^2^ Mental Health Institut de Recerca Sant Pau (IR Sant Pau) Barcelona Spain; ^3^ Hospital de la Santa Creu i Sant Pau Barcelona Spain; ^4^ Centro de Investigación Biomédica en Red Salud Mental (CIBERSAM) Instituto de Salud Carlos III (ISCIII) Madrid Spain

**Keywords:** addiction severity, cocaine use disorder, cocaine withdrawal symptoms, emotion regulation, hospitalization

## Abstract

**Objective:**

Traditional treatment approaches in cocaine use disorder (CUD) still report high rates of poor outcomes. Emotion regulation (ER) is a transdiagnostic factor that may contribute to the initiation and maintenance of CUD, strengthening addiction severity. A deeper understanding of ER in this population is crucial for improving treatment outcomes. This study explores whether ER difficulties at treatment entry predict addiction severity and withdrawal symptom severity after discharge in patients with CUD. Secondarily, it examines whether withdrawal symptom severity mediates the relationship between ER difficulties and relapse.

**Methods:**

A total of 70 CUD patients underwent a 14‐day inpatient detoxification. At admission, cocaine use–related variables and ER questionnaires (DERS and ERQ) were registered. After discharge, addiction severity (SDS), craving (WCS), cocaine withdrawal symptom severity (CSSA) and relapse were recorded. Multiple linear regression and mediation analysis were conducted to address the primary and secondary aims, respectively. This study draws on data from a larger randomized clinical trial.

**Results:**

Impulse control difficulties and nonacceptance of emotional responses predicted SDS, and nonacceptance of emotional responses predicted CSSA. However, mediation analyses showed no indirect effect of DERS total score on relapse through the effect of the mediating variable CSSA.

**Conclusion:**

ER screening at hospitalization admission may optimize treatment by identifying high‐risk CUD patients. Third‐generation therapies targeting ER skills may enhance outcomes by helping patients manage emotional distress, potentially reducing addiction severity and withdrawal symptoms. Because of mixed results and the exploratory nature of this study, further research on the ER role in this context is needed.

**Trial Registration:** ClinicalTrials.gov; ID: NCT05207228.

## Summary

- Nonacceptance of emotional responses at inpatient entry predicted severity of cocaine withdrawal symptoms following hospital discharge.
- Screening for emotion regulation difficulties at treatment entry could help to identify cocaine users who are at a higher risk of developing a more severe addiction.
- A paradigm shift in clinical practice may be beneficial, moving beyond traditional detoxification‐focused models towards interventions that also prioritize emotional skill‐building, emotional acceptance and behavioural modification.
- Further research is needed to better explore the role of emotion regulation in treatment outcomes among cocaine users by extending this study to different settings.

## 1 Introduction

Cocaine use is highly distressing for users and their environment (Clinical Guidelines on Drug Misuse and Dependence Update 2017 Independent Expert Working Group 2017; Kober 2014; World Health Organization and United Nations Office on Drugs and Crime 2020). The average interval between first cocaine use and treatment entry is 13 years, which increases the likelihood of developing drug‐related mental health issues given the highly addictive nature of cocaine (European Monitoring Centre for Drugs and Drug Addiction [EMCDDA] 2024).

Emotions are adaptive responses to specific needs required to achieve homeostasis. However, extreme emotions—positive and negative—can interfere with decision‐making and rational processing, promoting impulsive, risky behaviours like drug use (Cyders and Smith 2008; Tice et al. 2001). The urgency trait, or the propensity to act impulsively under intense affect, reinforces these actions as coping strategies, impairing emotion regulation (ER) (Cyders and Smith 2008). Additionally, drug use exacerbates pre‐existing psychiatric comorbidities, including mood, anxiety and stress‐related disorders (Brady and Sinha 2005; Clinical Guidelines on Drug Misuse and Dependence Update 2017 Independent Expert Working Group 2017), further impairing emotional processing (Dingle et al. 2018).

According to Gratz and Tull (2010), ER is the ability to face extreme emotions adaptively rather than avoiding or suppressing them (Sloan et al. 2017). Conceptualized as a multidimensional construct, ER encompasses: (1) awareness, understanding and acceptance of emotions; (2) goal‐directed behaviours and impulse control during distress; (3) cognitive flexibility to modulate frequency, duration and intensity of negative emotions; and (4) disposition to experience adverse emotions to enhance performance and have meaningful experiences (Gratz and Roemer 2004; Gratz and Tull 2010). Deficits in these abilities, termed emotion dysregulation, constitute a transdiagnostic factor involved in the aetiology and maintenance of diverse mental health conditions, including substance use disorders (SUD) (Berking and Wupperman 2012; Garke et al. 2021; González‐Roz et al. 2024; Gratz and Tull 2010; Shadur and Lejuez 2015; Stellern et al. 2022; Weiss et al. 2022).

Different models have been developed to conceptualize the underlying mechanisms that predispose individuals to develop cocaine use disorder (CUD). The affective processing model of negative reinforcement suggests that the lack of certain ER abilities may lead to substance use as a maladaptive coping mechanism to reduce or alleviate adverse emotions when facing distressing events (Baker et al. 2004; Weiss et al. 2022). Likewise, the stress‐based model of addiction posits that repeated stress exposure, together with genetic and environmental vulnerability, can lead to adaptations in biological stress and reward systems that increase the frequency of drug use and craving (Brady and Sinha 2005).

Traditional substance use psychotherapy approaches have focused on achieving abstinence and preventing relapses, targeting craving and withdrawal symptoms management. Even so, this approach still presents poor treatment outcomes, with high dropout and relapse rates (Dingle et al. 2018). Incorporating ER may enhance treatment outcomes in SUD, particularly in CUD, due to its relevance in the onset and maintenance of the addiction (Stellern et al. 2022; Weiss et al. 2022). This is especially relevant during inpatient detoxification treatment, a critical stage in the recovery process where extreme emotions arise due to substance withdrawal. Given the current evidence, prevailing approaches do not include ER in treatment during this stage (Dingle et al. 2018; Fox et al. 2007; Garke et al. 2021).

Most studies on ER and substance use have been conducted in nonclinical samples (Weiss et al. 2022). Similarly, recent systematic reviews of ER in SUD have primarily included studies of patients whose principal substance was alcohol, not cocaine (Stellern et al. 2022; Weiss et al. 2022), making it difficult to apply those findings to patients needing treatment for cocaine use.

Major gaps remain in understanding ER's role in CUD. The studies conducted to date have primarily examined how ER‐related variables affect treatment outcomes (e.g., frequency of use, post‐treatment abstinence and dropout). Craving and cocaine withdrawal symptoms severity are well‐established predictors of treatment outcomes, regardless of addiction severity (Ahmadi et al. 2006; Bisaga et al. 2010; Crits‐Christoph et al. 2007; Kampman et al. 2002; Palazón‐Llecha et al. 2024; Siqueland et al. 2002). At present, the role of ER in addiction severity, craving and cocaine withdrawal symptoms severity is not well understood (Decker et al. 2016; Fox et al. 2007; Garke et al. 2021; Weiss et al. 2022). Thus, more data are needed to better characterize how emotion dysregulation contributes to the maintenance of CUD.

In recent years, a growing number of studies have evaluated ER as a transdiagnostic mechanism in SUD. Nonetheless, which specific ER abilities and strategies underlie cocaine dependence remains poorly understood. Further research may determine whether ER‐related variables predict addiction severity, craving, and/or withdrawal symptoms severity in cocaine users to optimize treatment (Weiss et al. 2022).

### 1.1 Aim of the Study

In this context, this study mainly aimed to explore whether ER abilities and strategies assessed at hospital admission predict addiction severity, craving and withdrawal symptoms severity after discharge in CUD patients. A secondary aim was to test the presence of an indirect or direct effect of whether cocaine withdrawal symptoms severity post‐discharge mediates the association between ER difficulties at treatment entry and relapse. Assessing ER‐related variables at treatment entry could provide valuable data to improve hospitalization for detoxification treatment, as better characterize addiction course and prognosis after discharge.

## 2 Materials and Methods

### 2.1 Participants

At baseline, the sample included 70 participants (27 women) between the ages of 21 and 64 years (M = 42.9 years; SD = 8.2) hospitalized for cocaine detoxification. At the second assessment, the sample was composed of 65 participants, as five participants voluntarily discontinued treatment and were subsequently withdrawn from the study.

The study inclusion criteria were as follows: (a) being hospitalized for cocaine detoxification treatment; (b) abstinent at the first study assessment (3 days after hospital admission); (c) age ≥ 18 years.

Exclusion criteria were as follows: (a) treatment indication for reasons other than cocaine detoxification; (b) presence of severe mental disorder or neuropsychological alterations that could affect study participation; (c) opioid abuse or dependence during the last year; (d) insufficient knowledge of Spanish or Catalan language, or difficulties in reading or writing that could hinder participation; (e) refusal to accept the study procedures and/or sign the informed consent form.

### 2.2 Procedures

This clinical longitudinal study was conducted in accordance with the Declaration of Helsinki (1964) and approved by the Institut de Recerca Sant Pau ethical committee (IIBSP‐CTB‐2020‐116). This study used data from a randomized clinical trial (RCT) performed at our institution (ClinicalTrials.gov; ID: NCT05207229). The RCT evaluated the effects of a web‐based cognitive‐behavioural intervention (CBT4CBT) on treatment outcomes in patients with CUD. Participants who completed the 14‐day hospitalization for detoxification subsequently entered the 8‐week outpatient treatment, during which they received treatment as usual (TAU), consisting of day treatment and weekly individual sessions. Some were randomized to also receive CBT4CBT (TAU + CBT4CBT). Further details are available elsewhere (Mallorquí‐Bagué et al. 2023).

Specifically, data for the current study were collected from two assessments performed 48–72 h post‐hospitalization (first assessment) and 72 h after discharge from the 14‐day detoxification treatment (second assessment).

Data collection at each assessment was conducted in the presence of a trained clinical researcher, who reviewed all questionnaire items to minimize missing data. All study data were collected and stored in Clinapsis (clinapsis‐estudis.com) and then exported to the IBM‐SPSS software for statistical analysis. The data that support the findings of this study are available on request from the corresponding author. Data are not publicly available due to privacy or ethical restrictions.

All patients admitted to our addiction unit for cocaine detoxification were screened for possible inclusion in the present study. At hospitalization entry, patients who met the inclusion criteria were invited to participate when acute intoxication and withdrawal symptoms were no longer present. Those who agreed to participate were required to sign an informed consent form and were asked to complete the treatment entry evaluation.

The assessment consisted of a battery of questionnaires to assess the following: history of drug use; cocaine use variables; addiction severity; craving; severity of cocaine withdrawal symptoms; psychopathology; behavioural addictions; and ER‐related variables.

Three days (72 h) after discharge from the 14‐day inpatient cocaine detoxification programme, participants were asked to complete the second assessment, which included the same variables evaluated in the first assessment (except for history of drug use).

The primary focus of the present study was to evaluate the association between ER‐related variables at hospital admission and addiction severity, craving and severity of cocaine withdrawal symptoms after treatment discharge. The participants in the clinical trial completed a battery of questionnaires; however, here we describe only those relevant to the current study aims and administered at the two study time points (at treatment entry and at 72 h post‐discharge).

The secondary objective of this study aims to test the natural direct and indirect effects of the mediation model. Given that withdrawal symptoms severity is a well‐established predictor of poor treatment outcomes, the mediation model assumes that greater withdrawal symptoms severity after detoxification discharge will predict relapse after inpatient treatment discharge. Furthermore, ER difficulties at treatment entry may reduce tolerance to emotional distress, which, through heightened withdrawal severity after hospitalization discharge, can increase the likelihood of relapse into cocaine use after detoxification discharge. Nevertheless, craving levels after hospitalization discharge and treatment engagement are considered important confounders that could explain the effect of the mediator on the outcome, since evidence links them to poor treatment outcomes. The AGReMA‐SF Statement was used to guide the report of the mediation analyses procedures and outcomes (Lee et al. 2021).

Sociodemographic data were obtained at hospital admission. Cocaine use–related variables were obtained at hospital admission and at discharge. Treatment outcomes, including dropout and relapse rates, were assessed 3 days after treatment discharge.

During inpatient detoxification, participants received personalized psychiatric care for withdrawal and psychopathological symptoms, together with nursing care. Nurses were responsible for leading psychoeducation groups about drug use and relapse prevention. They also collected urine samples for urinalyses. Daily psychological treatment was delivered by trained psychologists (the primary investigators in this study) only to the participants who were included in the study.

The psychological intervention was based on a CBT approach that focused on motivation for change, craving recognition and management, assertiveness training and problem‐solving strategies.

### 2.3 Instruments

#### 2.3.1 Sociodemographic Data

Collected data included: age; sex (male/female); marital status (single/married/divorced/widow); and years of education.

#### 2.3.2 Cocaine Use–Related Variables

At treatment entry, the following data were registered: number of days of cocaine use in the previous 30 days; amount of cocaine per occasion; number of days since the last cocaine use; administration route (oral, intranasal, pulmonary, intravenous or other); and benzoylecgonine value. At post‐discharge, dropout and relapse rates, days since last cocaine use and benzoylecgonine value were registered. The Kinetic Microparticle Interaction test (Roche Diagnostic Systems Inc., Sommerville, NJ, USA) was used to check for the presence of the cocaine metabolite benzoylecgonine in urine at treatment entry and at discharge.

#### 2.3.3 Treatment Outcomes

Dropout and relapse rates were determined as dichotomous variables (yes/no) 3 days post‐discharge. Relapse was assessed through urinalyses, with the test considered positive for cocaine use when benzoylecgonine levels were ≥ 300 μg/L. Participants who failed to attend the Day 3 visit and did not respond to the researchers telephonic calls were classified as dropouts.

#### 2.3.4 Difficulties in Emotion Regulation Scale (DERS)

The Spanish DERS version, validated with good psychometric properties, was used as a self‐report instrument to assess ER problems (Hervás and Jódar 2008). The scale comprises six subscales, rated on a 5‐point Likert scale: nonacceptance of emotional responses; difficulty in engaging in goal‐directed behaviour; impulse control difficulties; lack of emotional awareness; limited access to ER strategies; and lack of emotional clarity. Higher scores (both overall and on each subscale) indicated greater difficulties with ER. For the present study, the Cronbach alpha value for the total DERS was 0.90. By subscale, the Cronbach alpha values were as follows: impulse control difficulties, 0.77; difficulty engaging in goal‐directed behaviours, 0.72; lack of emotional awareness, 0.81; lack of emotional clarity, 0.74; nonacceptance of emotional responses, 0.88; and limited access to ER strategies, 0.82.

#### 2.3.5 Emotion Regulation Questionnaire (ERQ)

The ERQ was used to assess two ER strategies—cognitive reappraisal and expressive suppression—on a 7‐point Likert scale. Higher scores indicate greater use of these ER strategies. For the present study, the Spanish adaptation of the ERQ was used, which has demonstrated satisfactory psychometric properties (Cabello et al. 2013). ERQ data obtained at treatment entry were used in the analysis. The internal consistency of the two subscales was *α* = 0.80.

#### 2.3.6 The Severity of Dependence Scale (SDS)

The SDS was used to assess substance dependence severity through a 5‐item measure on a 4‐point Likert scale, with higher scores reflecting greater dependence. For the post‐discharge assessment, participants were asked to assess the previous 24 h. The Spanish validation study showed optimal psychometric properties for this scale (González‐Sáiz and Salvador‐Carulla 1998). In the current study, the internal consistency of this scale was *α* = 0.50.

#### 2.3.7 Cocaine Selective Severity Assessment (CSSA)

The 18‐item CSSA was used to assess cocaine withdrawal symptoms on a 7‐point Likert scale, with a higher total score suggesting greater severity. Although some of the psychometric properties of the Spanish validation are satisfactory, the scale has some limitations that should be considered (Pérez de los Cobos et al. 2014). In the current study, this scale showed adequate internal consistency (*α* = 0.80).

#### 2.3.8 Weiss Craving Scale (WCS)

The Spanish version of the WCS is a five‐item, 9‐point Likert scale used to measure craving in cocaine users and has displayed adequate psychometric properties. Higher scores indicate greater craving (Tejero et al. 2003). For the present study, WCS data were obtained from the postdischarge assessment and demonstrated good internal consistency (*α* = 0.86).

### 2.4 Study Variables

The DERS and ERQ measure a range of ER‐related variables (abilities and strategies). These variables were evaluated as possible predictors of the dependent variables (addiction severity, craving and severity of cocaine withdrawal symptoms).

For the mediation analyses (secondary objective), the exposure variable was ER, assessed through DERS at the first assessment, once withdrawal symptoms were over. Severity of cocaine withdrawal symptoms, which is the mediation variable, was measured using the CSSA questionnaire at the second assessment, 72 h after hospitalization discharge. The outcome variable, relapse, was collected at the second assessment through urinalysis. Relapse was considered positive when benzoylecgonine values were > 300 μg/L. Craving levels assessed through the WCS, at the second assessment and treatment engagement, which was an unmeasured variable, were considered potential confounders that may explain the effect of the mediator on the outcome. The detailed properties of each measurement tool can be found above, in the Instruments subsection. Additionally, no blinded assessment was used in any case.

### 2.5 Statistical Analyses

The IBM‐SPSS statistical software package (SPSS Inc. Chicago, IL, USA) was used to perform the statistical analysis. The hypothesis tests were two‐tailed (*α* = 0.05). Descriptive measures, including mean, standard deviation (SD), range and frequency distributions, were obtained for sociodemographic data and cocaine use–related variables. The Kolmogorov–Smirnov test was used to check the normality assumption. T‐tests for paired samples were performed to explore differences between cocaine use variables, ER‐related variables and addiction severity, craving and severity of cocaine withdrawal symptoms at treatment entry and at discharge.

To perform the linear regression analyses, Pearson correlations were used to examine bivariate relationships between post‐discharge scores on the three scales (SDS, WCS and CSSA) and the following variables: years of cocaine use; days of cocaine use in the last 30 days; daily amount of cocaine; days since last cocaine use (both at treatment entry and after discharge); benzoylecgonine levels at treatment entry and after discharge; DERS subscale scores and total score at treatment entry and the two ERQ subscales at treatment entry. For multivariate analyses, variables that were significantly (*α* = 0.05) associated with the DERS subscales on the bivariate analyses were considered candidate predictors and included in the linear regression analyses.

Multiple linear regression analyses were performed using a backward stepwise method to explore whether ER‐related variables at treatment entry predicted addiction severity, craving, and/or severity of cocaine withdrawal symptoms after treatment discharge. Even though the stepwise regression methods have been often criticized, they seem to endure as adequate and valuable exploratory data analysis techniques and are therefore well suited in exploratory studies (Lomax 2001; Wittink 1988) like the current one. Moreover, the superiority of the backward selection procedure over other stepwise variable selection procedures is well documented (Darlington and Hayes 2017).

A counterfactual‐based framework was used for mediation analyses, with bootstrapping as the specific method for conducting the analyses (UCLA: Statistical Consulting Group 2024). The PROCESS v.4.2 plug‐in for IBM SPSS v.25 was used to perform the mediation analyses. Linear regression was used to model the association between ER difficulties (DERS total score) and withdrawal symptoms severity (CSSA), and binary logistic regression was used to model both the relationship between CSSA and relapse, and the direct effect of DERS total score on relapse. A linear relation was assumed between DERS total score and CSSA, as well as between predictors and the log‐odds of relapse. No interaction terms were included. A backward stepwise selection method was used to identify craving levels after hospitalization discharge as a potential confounder of the exposure–mediator, exposure–outcomes and mediator–outcome associations. Although treatment engagement may be a potential confounder, it was unmeasured. No imputation methods were used to handle missing data, as most of the missingness resulted from participants who voluntarily discontinued treatment.

## 3 Results

### 3.1 Descriptive

Overall, the clinical profile of this sample exhibits a mean (SD) of 16.3 (9.8) years of cocaine use and a mean (SD) consumption frequency of 18 (9.6) days per month. The mean amount of cocaine used per occasion was 1202.9 (1098.8) mg and the mean time since last consumption was 6 (8.5) days, both at the first assessment. In most cases (*n* = 59, 86.8%), the route of administration was intranasal. Table 1 shows the detailed clinical and sociodemographic data.

**TABLE 1:** Descriptive statistics, including means (M) and standard deviations (SD), are presented for sociodemographic variables and cocaine use–related variables.

	*N*	Range	M (SD)		*n* (%)
**Sociodemographic variables**					
Age	70	21–64	42.9 (8.2)		
Schooling years	63	6–26	11.6 (4.1)		
Sex	70				
Male					43 (61.4)
Female					27 (38.6)
Marital status	68				
Single					28 (41.2)
Married					19 (27.9)
Divorced					21 (30.9)
Widowed					0
Route of cocaine administration	68				
Intranasal					59 (86.8)
Pulmonary					8 (11.8)
Other					1 (1.5)
Dropout after treatment discharge	70				
Yes					7 (10)
No					63 (90)
Relapse after treatment discharge	65				
Yes					10 (15.4)
No					55 (84.6)
**Cocaine use**–**related variables**					
Benzoylecgonine level at treatment entry, μg/L	66	40–1000	862.1 (329.8)		
Benzoylecgonine level after treatment discharge, μg/L	56	40–1000	101.7 (222.6)		
Years of cocaine use	70	1–44	16.3 (9.8)		
Days of cocaine use last 30 days	70	0–30	18 (9.6)		
Daily cocaine amount, mg	68	0–6000	1202.9 (1098.8)		
Days since last cocaine use at treatment entry	69	0–67	6.1 (8.5)		
Days since last cocaine use after treatment discharge	65	1–74	18 (11.1)		

Statistically significant reductions between treatment entry and the post‐discharge assessment were observed in ER‐related variables, cocaine use, addiction severity, craving and severity of cocaine withdrawal symptoms. The size effect ranged from small to large. The most significant changes were observed in benzoylecgonine levels (*t* = 15.7; *p* < 0.001; *d* = 2.1), in the number of days since the last cocaine use (*t* = −11.83; *p* < 0.001; *d* = 1.5), and in SDS scores (*t* = 7.75; *p* < 0.001; *d* = 1).

We observed decreases in the expressive suppression subscale of the ERQ (*t* = 2; *p* = 0.05; *d* = 0.3) and in the following DERS subscales: impulse control (*t* = 3.82; *p* < 0.001; *d* = 0.5), nonacceptance of emotional responses (*t* = 2.96; *p* = 0.004; *d* = 0.4), access to ER strategies (*t* = 2.58; *p* = 0.012; *d* = 0.3) and DERS total score (*t* = 3.09; *p* = 0.003; *d* = 0.4), with a small effect size. Decreases in WCS (*t* = 4.66; *p* < 0.001; *d* = 0.6) and CSSA (*t* = 3.84; *p* < 0.001; *d* = 0.5) scores showed a moderate effect size. The full results are shown in Table 2.

**TABLE 2:** Mean scores (M), standard deviations (SD) and differences in mean scores obtained at treatment entry and after discharge for cocaine use variables, ER‐related variables, addiction severity, craving and severity of cocaine withdrawal symptoms.

	Treatment entry	After treatment discharge	*t*	*p*	*d*
	M (SD)	M (SD)			
Benzoylecgonine in urine, μg/L	881 (311.5)	103.7 (226.5)	15.67	< 0.001**	2.1
Days since last cocaine use	6 (8.7)	17.8 (11.1)	−11.83	< 0.001**	1.5
DERS impulse control difficulties	16.9 (5.4)	14.4 (6.2)	3.82	< 0.001**	0.5
DERS difficulties in goal‐directed behaviours	16.9 (4.2)	15.8 (5)	1.96	0.055	0.3
DERS lack of emotional awareness	18.3 (5.6)	17.5 (5.4)	1.25	0.21	0.2
DERS lack of emotional clarity	13.1 (4.5)	12.3 (5.3)	1.57	0.12	0.2
DERS nonacceptance	18.2 (6.9)	15.9 (7.1)	2.96	0.004**	0.4
DERS limited access to ER strategies	23.1 (7)	20.4 (7.9)	2.58	0.012*	0.3
DERS total	105 (22.3)	96.4 (28.1)	3.09	0.003**	0.4
ERQ cognitive reappraisal	27 (7.4)	25.9 (7.5)	0.88	0.38	0.01
ERQ expressive suppression	17.4 (7)	15.8 (6)	2	0.050*	0.3
Addiction severity (SDS)	9.6 (2.4)	6.1 (2.9)	7.75	< 0.001**	1
Craving (WCS)	3.4 (2.6)	1.6 (2.1)	4.66	< 0.001**	0.6
Severity of cocaine withdrawal symptoms (CSSA)	32 (18.8)	21.9 (17.4)	3.84	< 0.001**	0.5

Abbreviations: CSSA, Cocaine Selective Severity Assessment; DERS, Difficulties in Emotion Regulation Scale; ERQ, Emotion Regulation Questionnaire; SDS, Severity of Dependence Scale; WCS, Weiss Craving Scale.

* *p* ≤ 0.05.

** *p* ≤ 0.01.

### 3.2 Correlations Between Cocaine Use–Related Variables, Addiction Severity, Severity of Cocaine Withdrawal Symptoms, Craving and ER‐Related Variables

We assessed correlations (Pearson's) between the DERS variables obtained at treatment entry and the SDS, WCS and CSSA scales administered after hospital discharge. CSSA was moderately correlated with all of the DERS variables: impulse control difficulties (*r* = 0.31; *p* = 0.020), nonacceptance of emotional responses (*r* = 0.33; *p* = 0.013), difficulties in accessing ER strategies (*r* = 0.34; *p* = 0.012) and the DERS total score (*r* = 0.39; *p* = 0.004). SDS, by contrast, was only correlated with a single ER‐related variable, nonacceptance of emotional responses, and this correlation was weak (*r* = 0.25; *p* = 0.048).

The number of days of cocaine use in the last 30 days at treatment entry was moderately associated with post‐discharge scores on the WCS (*r* = 0.30; *p* = 0.015) and with the number of days since last cocaine use at treatment discharge (*r* = −0.30; *p* = 0.15). The number of days since last cocaine use at treatment entry was weakly associated with impulse control difficulties (*r* = −0.26; *p* = 0.035) and with cognitive reappraisal (*r* = 0.30; *p* = 0.015) at the time of hospitalization. Detailed results are shown in Table 3.

**TABLE 3:** Correlations between cocaine use–related variables, addiction severity, severity of withdrawal symptoms, craving and ER‐related variables.

	1	2	3	4	5	6	7	8	9	10	11	12	13	14	15	16	17	18	19
1. Years of cocaine use	—																		
2. Days of cocaine use last 30 days	−0.09	—																	
3. Daily cocaine amount (mg)	0.02	0.14	—																
4. Days since last cocaine use at entry	0.16	−0.41**	−0.07	—															
5. Days since last cocaine use after discharge	0.15	−0.30*	−0.05	0.70**	—														
6. Benzoylecgonine level (μg/L) at entry	−0.21	0.33**	−0.03	−0.54**	−0.48**	—													
7. Benzoylecgonine level (μg/L) after discharge	0.13	0.09	0.07	−0.001	−0.22	0.11	—												
8. DERS impulse control difficulties at entry	−0.19	0.16	−0.07	−0.26*	0.02	0.17	−0.22	—											
9. DERS difficulty engaging goal‐directed behaviours at entry	−0.08	−0.05	−0.13	−0.02	0.06	−0.01	−0.12	0.5**	—										
10. DERS lack of emotional awareness at entry	0.25*	−0.20	0.09	−0.03	0.12	0.15	−0.17	0.002	−0.22	—									
11. DERS Lack of emotional clarity at entry	0.14	−0.01	0.22	−0.02	0.09	−0.04	0.00	0.19	0.03	0.60**	—								
12. DERS nonacceptance of emotional responses at entry	−0.16	0.11	−0.20	−0.02	−0.02	−0.14	0.01	0.51**	0.58**	−0.14	0.23	—							
13. DERS limited access to emotion‐regulation strategies at entry	−0.09	0.08	−0.05	0.02	0.12	−0.09	−0.09	0.73**	0.64**	−0.09	0.24	0.72**	—						
14. DERS total score at entry	−0.03	0.05	−0.04	−0.07	0.10	−0.02	−0.14	0.80**	0.67**	0.28*	0.59**	.77**	0.86**	—					
15. ERQ cognitive reappraisal at entry	−0.001	0.08	−0.04	0.30*	0.21	−0.13	0.09	−0.16	0.07	−0.38**	−0.19	0.06	−0.11	−0.18	—				
16. ERQ expressive suppression at entry	0.09	0.12	0.26*	0.03	0.09	−0.18	−0.15	−0.02	−0.13	0.17	0.36**	0.09	0.07	0.16	0.33**	—			
17. SDS after discharge	−0.12	0.07	−0.11	−0.20	−0.36**	0.20	0.26	0.05	0.19	−0.24	−0.14	0.25*	0.18	0.11	−0.06	−0.14	—		
18. CSSA after discharge	−0.21	0.02	−0.04	−0.14	−0.18	0.16	0.26	0.31*	0.25	0.04	0.21	0.33*	0.34*	0.39**	0.09	−0.10	0.39**	—	
19. WCS after discharge	−0.13	0.30*	−0.18	−0.13	−0.17	0.21	0.30*	0.16	0.13	−0.008	0.13	0.20	0.16	0.22	0.18	−0.01	0.34**	0.61**	—

Abbreviations: CSSA, Cocaine Selective Severity Assessment; DERS, Difficulties in Emotion Regulation Scale; ERQ, Emotion Regulation Questionnaire; SDS, Severity of Dependence Scale; WCS, Weiss Craving Scale.

* *p* ≤ 0.05.

** *p* ≤ 0.01.

### 3.3 ER Abilities as Predictors of Addiction Severity and Severity of Cocaine Withdrawal Symptoms

On the multivariate linear regression analyses, two DERS subscales (impulse control difficulties and nonacceptance of emotional responses) at treatment entry were significant predictors of SDS after treatment discharge (*R* ^2^ = 0.13; *F*_(2,57)_ = 4.1; *p* = 0.022). Nonacceptance of emotional responses at treatment entry was a significant predictor of CSSA scores at treatment discharge (*R* ^2^ = 0.13; *F*_(1,52)_ = 7.9; *p* = 0.007). None of the DERS subscales were predictive of craving at treatment discharge and therefore the craving‐related results are not shown in the table. Table 4 shows the results of these analyses.

**TABLE 4:** Predictors of addiction severity and severity of cocaine withdrawal symptoms.

Criteria	Predictors	*B*	SE	*t*	*R* ^2^	*F*_(df)_	*p*	*f* ^2^	1 − *β*
SDS after treatment discharge	DERS impulse control difficulties at treatment entry	−0.14	0.08	−1.75	0.13	4.1_(2,57)_	0.022	0.14	0.80
	DERS nonacceptance of emotional responses at treatment entry	0.18	0.06	2.85**					
CSSA after treatment discharge	DERS nonacceptance of emotional responses at treatment entry	0.91	0.32	2.81**	0.13	7.9_(1,52)_	0.007	0.15	0.80

Abbreviations: CSSA, Cocaine Selective Severity Assessment; DERS, Difficulties in Emotion Regulation Scale; SDS, Severity of Dependence Scale.

** *p* ≤ 0.01.

### 3.4 Severity of Cocaine Withdrawal Symptoms as a Mediating Variable

Multiple linear regression analyses were performed to explore whether the severity of cocaine withdrawal symptoms mediates the association between difficulties in ER and treatment outcomes, especially relapse. Data regarding baseline characteristics of the participants included in the mediation analyses are detailed in Supporting Information S1.

**FIGURE 1:**
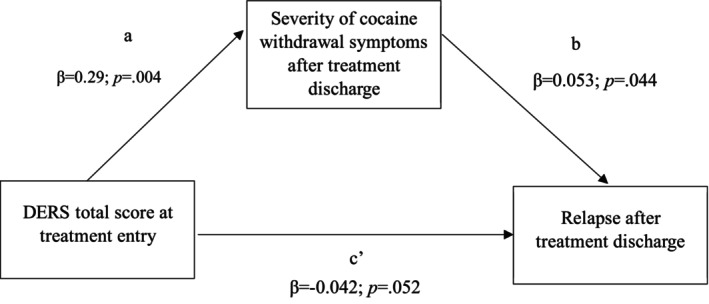
Mediation model showing severity of cocaine withdrawal symptoms after treatment discharge as a possible mediation factor between DERS total score at treatment entry and relapse after treatment discharge. *Note:* Direct effect: −0.042 [95% CI: −0.084 to 0.0004]. Indirect effect: 0.015 [95% CI: −0.007 to 0.061]. Abbreviations: *DERS*, Difficulties in Emotion Regulation Scale; *a*, regression coefficient of *X* on *M*; *b*, regression coefficient of *M* on *Y*; *c′,* regression coefficient of *X* on *Y*; *p*, *p* value; *β*, regression coefficient; CI, confidence interval.

According to the mediation model, the DERS total score was a significant predictor of CSSA (*b* = 0.28; *t* = 3.01; *p* = 0.004; *R* ^2^ = 0.15), and CSSA significantly predicted relapse (*b* = 0.05; z = 2.01; *p* = 0.044; *R* ^2^ = 0.18). However, the direct effect of the DERS total score on relapse was not statistically significant (*b* = −0.042; z = −1.94; *p* = 0.052; *R* ^2^ = 0.18). The indirect effect of the DERS total score on relapse through CSSA was estimated using bootstrapped confidence intervals (CI). The effect was not significant (indirect effect = 0.015; 95% CI: −0.007 to 0.061), indicating that CSSA did not mediate the relationship between the DERS total score and relapse (Figure 1). A continuous scale using unstandardized coefficients was employed to estimate effects.

## 4 Discussion

ER allows one to face adverse emotional states by modifying behaviour (functional adaptation) instead of controlling those emotional states by suppressing or avoiding them (Gratz and Roemer 2004; Gratz and Tull 2010). Although ER has been established as a transdiagnostic mechanism in other psychopathological conditions, including substance use disorders (Berking and Wupperman 2012; Garke et al. 2021; Stellern et al. 2022; Weiss et al. 2022), its role in CUD remains unclearly defined (Weiss et al. 2022). Therefore, this study was conducted to better characterize the role of ER in CUD.

The main finding of this study is that difficulties in two ER abilities—impulse control and nonacceptance of emotional responses—significantly predicted addiction severity after discharge. Participants with greater impulse control difficulties had less severe addiction at post‐discharge than those better able to control those impulses. These findings do not align with previous reports indicating that heavier cocaine users have reduced emotional flexibility (defined as the inability to avoid or change impulsive behaviour under emotional distress) (Fox et al. 2007; Tice et al. 2001; Verdejo‐García et al. 2007; Weiss et al. 2022).

Studies show that impulsive behaviours used to alleviate distressing emotions reinforce addictive behaviours, undermining treatment effectiveness, especially in residential treatment programmes (Fox et al. 2007; Garke et al. 2021). Impulse control difficulties, often linked to trait urgency, increase risk behaviours and serve as coping strategies in those facing intense emotions, impairing decision‐making and rational processing systems, contributing to substance dependence (Fox et al. 2007; Garke et al. 2021; González‐Roz et al. 2024; Verdejo‐García et al. 2007). Our findings show that nonacceptance of emotional responses contributes to addiction severity, consistent with previous reports showing that substance‐related cues can trigger negative affective reactivity in cocaine users by increasing a judgmental and rejection response towards this emotional state. Difficulties in developing acceptance and tolerance attitudes towards distressing emotions impede the individual's capacity to modify behaviour in response to these emotional states, maintaining cocaine use (Cavicchioli et al. 2019; Gratz and Tull 2010; Witkiewitz et al. 2013).

Nonacceptance of emotional responses predicted the severity of cocaine withdrawal symptoms (see Table 4), suggesting that those unable to accept their emotional responses experience more severe cocaine withdrawal symptoms after hospital discharge, which has previously been described as a predictor of adverse outcomes in cocaine dependence (Ahmadi et al. 2006; Kampman et al. 2002; Palazón‐Llecha et al. 2024).

Considering the main findings, we conducted mediation analyses to explore whether withdrawal symptoms severity (CSSA score post‐discharge) mediates the relation between ER difficulties (DERS total score at treatment entry) and relapse. As Figure 1 shows, ER difficulties at treatment entry did not predict relapse, nor did withdrawal symptoms severity mediate their relationship. These findings could be interpreted within the negative reinforcement model, which states that emotional dysregulation promotes drug use to alleviate cocaine withdrawal symptoms (Garke et al. 2021; Shadur and Lejuez 2015; Volkow et al. 2016), thus increasing use frequency (Dingle et al. 2018). However, our results only partially support this model. As expected, greater difficulties in ER abilities at treatment entry predicted more severe cocaine withdrawal symptoms post‐discharge, and these, in turn, predicted relapse. Yet, unexpectedly, higher ER difficulties predicted a lower likelihood of relapse. This may be due to the second assessment occurring just 3 days post‐discharge, before randomization. That is, the assessment was performed in the context of the clinical trial, when participants were assumed to have resumed their daily routine. Perhaps this interval was too short to determine the true relapse rate as patients are more likely to remain abstinent in the days immediately following hospital discharge. Therefore, future studies—ideally longitudinal—should extend the postdischarge follow‐up. Given the short follow‐up period and specific clinical trial conditions, results should be interpreted cautiously when generalizing to other treatment settings.

In‐hospital interventions for cocaine detoxification typically combine pharmacological treatment and nursing care. In this study, participants received psychotherapy to promote abstinence and prevent relapse. Significant improvement in ER abilities (DERS subscales) was observed, especially in impulse control, nonacceptance of emotional responses, limited access to ER strategies, and the DERS total score. Improvements also occurred in benzoylecgonine levels, addiction severity, craving, cocaine withdrawal symptoms severity and days since last cocaine use. These results show that cocaine is used to cope with extreme emotional distress, seeking short‐term welfare, consistent with prior evidence (Baker et al. 2004; Weiss et al. 2022).

A small improvement was observed in the ERQ expressive suppression (but not in cognitive reappraisal). This, along with enhanced ER abilities and reduced cocaine dependence severity, indicates that the participant is better positioned to further develop ER abilities post‐hospitalization. Aligned with Fox et al., improvements in ER (DERS total score) were observed following inpatient treatment, particularly in accessing ER strategies, though impulse control difficulties persisted in CUD patients (Fox et al. 2007). Conversely, we found decreases in impulse control and acceptance difficulties, potentially reducing impulsive behaviours and enabling strategy access to manage emotional distress, thereby improving ER overall.

More days since last cocaine use post‐discharge moderately correlated with less severe addiction after discharge. Greater difficulty accepting emotional responses was weakly associated with higher addiction severity. Craving was moderately associated with the number of cocaine use days in the previous 30 days and with urinary benzoylecgonine levels post‐discharge, suggesting that more frequent pretreatment use and greater post‐discharge use sustain higher craving. Previous studies found that more cocaine use in the 30 days before treatment predicts worse outcomes (i.e., abstinence and treatment retention) post‐discharge (McKay et al. 2013; Palazón‐Llecha et al. 2024; Rash et al. 2008; Siqueland et al. 1998). Craving also predicts treatment failure and relapse (Bisaga et al. 2010; Crits‐Christoph et al. 2007). More severe cocaine withdrawal symptoms moderately correlated with greater ER difficulties, including impulse control, accessing ER strategies and emotional acceptance.

These findings support previous reports suggesting that ER is a transdiagnostic factor in the onset and maintenance of CUD, as shown in cocaine users (Fox et al. 2007), in studies with mixed cohorts (cocaine and other substances) (Garke et al. 2021; González‐Roz et al. 2024; Gratz and Tull 2010; Shadur and Lejuez 2015; Stellern et al. 2022; Weiss et al. 2022) and in other psychiatric conditions such as anxiety, depression, eating disorders and borderline personality disorder (Berking and Wupperman 2012; Vine and Aldao 2014; Sloan et al. 2017). Although all ER abilities are relevant for optimal emotion management, our findings suggest that difficulties in accepting emotional responses may be the most important addiction‐related ability, contributing to both addiction severity and withdrawal symptoms severity. Impulse control and emotional acceptance play a key role in CUD, particularly for addiction severity. Individuals lacking these may struggle to develop more complex ER abilities, like accessing strategies or engaging in goal‐directed behaviours. Unlike the negative reinforcement model, although pretreatment ER difficulties predicted postdischarge cocaine withdrawal symptoms severity, and symptoms severity predicted relapse, our results suggest that withdrawal symptoms severity did not mediate the association between ER difficulties and relapse.

This study has several limitations. First, as the main focus was to assess the impact of inpatient treatment, the findings may not generalize to other treatment settings (e.g., outpatient treatment), warranting future research on whether ER abilities also predict addiction and withdrawal symptom severity in outpatient CBT. Second, a large number of bivariate analyses have been conducted, which could be considered to increase the risk of type I errors in hypothesis‐driven studies. However, this is a widely accepted procedure for selecting potential predictor variables to include in subsequent regression analyses in exploratory studies. Consistently, the discussion focuses only on the results of the multivariable analyses. Third, self‐reported questionnaires were used. Fourth, although the psychotherapy delivered during hospitalization seems to enhance ER skills, the current design does not allow us to determine whether these changes would have occurred without this intervention. Further research is needed to determine its effectiveness. Fifth, due to the exploratory nature of most of the analyses carried out in the present study, the predictive validity of the models derived from multivariable regression analyses needs to be confirmed in other datasets. Sixth, in the mediation analyses, not considering treatment engagement, an unmeasured confounder, may have omitted a relevant explanatory mechanism underlying poor treatment outcomes, like relapsing into cocaine use. Additionally, relapse was assessed only 3 days post‐discharge, likely too soon for accurate detection, as abstinence often continues briefly after discharge. Concerning missing data, although only five participants (7.2%) voluntarily discontinued treatment, this may have influenced outcomes and affected the validity and reliability of the mediation model estimates. Finally, results must be considered in light of the sample size, which can limit statistical power and diminish the robustness and generalizability of all results.

To our knowledge, this is the first study to assess ER difficulties as potential predictors of addiction and withdrawal symptom severity in a sample comprised exclusively of CUD patients with extensive cocaine use histories, addressing a key gap in the literature. Unlike previous studies evaluating the role of ER‐related variables, this research was conducted in a clinical sample of hospitalized CUD patients undergoing detoxification, whereas most prior studies were performed in non‐clinical samples. Another strength is that we only included CUD patients, avoiding potential biases from mixed drug‐user samples, which can affect treatment response. Our aim was to minimize any potential bias related to the substance of abuse. Given the scarcity of studies on this topic, our study provides valuable insights into ER's predictive role in cocaine‐related variables. Another strength is that our results align with prior evidence supporting third‐generation therapies, reinforcing the validity of this treatment approach (Gratz and Tull 2010).

In terms of treatment implications, detoxification hospitalization offers a controlled setting where, once the substance is removed, patients may experience extreme and distressing emotions, facilitating ER skill development. According to the affective processing model of negative reinforcement and the stress‐based model of addiction, improved ER abilities shape coping styles and may protect against craving, withdrawal symptoms and other distressing events (Dingle et al. 2018; Fox et al. 2007; Garke et al. 2021). Incorporating ER skills training on a CBT approach once initial withdrawal symptoms have finished and patients can face distressing emotions could facilitate their management. Beyond behavioural modification, recognizing and accepting extreme emotions may enhance treatment efficacy by reducing addiction severity and cocaine withdrawal symptom severity. As ER deficits predict post‐treatment outcomes, these findings support shifting clinical practice beyond traditional detoxification‐focused models to include emotional skill‐building interventions.

Third‐generation therapies, such as acceptance and mindfulness‐based approaches, have demonstrated efficacy in improving impulse control, acceptance of emotional responses and other ER skills. The main mechanism of change involved adopting a nonevaluative attitude towards emotional experiences: emotion perceived as unfavourable leads to behavioural and emotional avoidance and suppression. Thus, learning to approach emotions without judgement is expected to decrease emotional reactivity (Gratz and Tull 2010).

Lastly, because ER predicts addiction severity post‐discharge and is a well‐known predictor of CUD treatment outcomes (Stellern et al. 2022; Weiss et al. 2022), our results highlight the need to screen for ER deficits at treatment entry. This may optimize inpatient care by targeting ER in patients with greater difficulties (Fox et al. 2007; Garke et al. 2021).

## 5 Conclusion

In conclusion, this is the first study to demonstrate ER deficits in cocaine users, with impulse control difficulties and acceptance of emotional responses predicting greater addiction severity and more severe cocaine withdrawal symptoms post‐discharge. These findings have two important treatment implications. First, assessing DERS before treatment could provide early insights into CUD prognosis, enabling more individualized interventions. Second, as impulse control and emotional acceptance difficulties reinforce ER's transdiagnostic role in CUD, our findings highlight the need to address ER deficits during inpatient treatment, beyond traditional approaches. Consistent with previous reports, our findings suggest that third‐generation therapies that develop core ER skills (e.g., accepting and modifying behaviour) may improve ER abilities and treatment outcomes. This approach equips patients to manage distressing emotional states more effectively, rather than attempting to control the emotional state through avoidance, emotional suppression, and/or rumination.

## Conflicts of Interest

The authors declare no conflicts of interest.

## Supporting information


**Data S1:** Supporting Information.

## Data Availability

The data that support the findings of this study are available on request from the corresponding author. Data are not publicly available due to privacy or ethical restrictions.
