# Supplementary material for: The Predictive Value of Emotion Regulation in Cocaine Use Disorder Severity: Psychotherapeutic Implications During Hospitalization for Detoxification
Source: Clin Psychol Psychother. 2025 Sep 22;32(5):e70155. doi: 10.1002/cpp.70155 (PMC12453906; doi:10.1002/cpp.70155)
Supplement: Supplementary file 1 — Data S1: Supporting Information. [file CPP-32-e70155-s001.docx]

**Supplementary material 1**

The analysis was conducted on a sample of 70 participants who underwent hospitalization for cocaine detoxification. The mean (SD) age of the participants was 42.9 (8.2) years, and 38.6% (n=27) were women. Participants reported a mean (SD) of 16.3 (9.8) years of cocaine use, and the mean baseline benzoylecgonine level (the metabolite of cocaine, considered positive at ≥300 ug/L) was 862.1 (329.8) ug/L. At baseline, the mean DERS total score was 105 (22.3) points, and the mean CSSA score was 32 (18.8) points. After treatment finalization, the mean CSSA score (used as the mediation variable) was 21.9 (17.4) points. Relapse, assessed three days after hospitalization discharge, was observed in 10 participants (15.4%). Detailed baseline and post-discharge assessment data are presented in Tables 1 and 2 in the manuscript.

During the follow-up phase, 5 participants voluntarily discontinued treatment before the outcome assessment (relapse). Among the remaining 65 participants, 7 of them had missing data for the mediator variable (CSSA after hospitalization for detoxification treatment discharge), and 5 had missing data for the selected confounder variable (WCS after hospitalization for detoxification discharge). A comparative analysis was conducted between participants with complete data and those with missing data during follow-up. No significant differences were found in age, sex, severity of cocaine withdrawal symptoms (CSSA), craving (WCS) or baseline DERS total score (Table 5).

**Table 5**

*Mean scores (M), standard deviations (SD) and differences in mean scores obtained at treatment entry for age, sex, severity of cocaine withdrawal symptoms, craving and DERS total score between participants with complete data and those with missing data.*

|  | Participants with complete data | Participants with missing data | t/*X^2^* | *p* | *d/V* |
| --- | --- | --- | --- | --- | --- |
|  | M (SD)/% (n) | M (SD)/% (n) |  |  |  |
| Age | 43.1 (8.4) | 40.2 (4.5) | .75 | .45 | .43 |
| Sex |  |  |  |  |  |
| *Male* | 57.1 (40) | 2.9 (2) | .9 | .34 | .11 |
| *Female* | 35.7 (25) | 4.3 (3) |  |  |  |
| Severity of cocaine withdrawal symptoms (CSSA) at baseline | 31.4 (18.3) | 23.5 (12.2) | .85 | .4 | .51 |
| Craving (WCS) at baseline | 3.4 (2.6) | 2.4 (2.2) | .81 | .42 | .41 |
| DERS total at baseline | 105.2 (22.9) | 107.5 (23.7) | -.2 | .85 | .1 |

^a^ *Note:* **p*≤.05; ***p*≤.01.

^b^ Abbreviations: *DERS,* Difficulties in Emotion Regulation Scale; *WCS,* Weiss Craving Scale, *CSSA,* Cocaine Selective Severity Assessment
